# Diversification of the ant odorant receptor gene family and positive selection on candidate cuticular hydrocarbon receptors

**DOI:** 10.1186/s13104-015-1371-x

**Published:** 2015-08-27

**Authors:** Patamarerk Engsontia, Unitsa Sangket, Hugh M. Robertson, Chutamas Satasook

**Affiliations:** Molecular Ecology and Evolution Research Unit, Prince of Songkla University, Songkla, 90112 Thailand; Department of Biology, Faculty of Science, Prince of Songkla University, Songkla, 90112 Thailand; Department of Molecular Biotechnology and Bioinformatics, Faculty of Science, The Center for Genomics and Bioinformatics Research, Prince of Songkla University, Songkla, 90112 Thailand; Department of Entomology, University of Illinois at Urbana-Champaign, 505 South Goodwin Avenue, Urbana, IL 61801 USA

**Keywords:** Insect odorant receptor, Molecular evolution, Leaf-cutter ant, Cuticular hydrocarbon, Positive selection

## Abstract

**Background:**

Chemical communication plays important roles in the social behavior of ants making them one of the most successful groups of animals on earth. However, the molecular evolutionary process responsible for their chemosensory adaptation is still elusive. Recent advances in genomic studies have led to the identification of large odorant receptor (*Or*) gene repertoires from ant genomes providing fruitful materials for molecular evolution analysis. The aim of this study was to test the hypothesis that diversification of this gene family is involved in olfactory adaptation of each species.

**Results:**

We annotated the *Or* genes from the genome sequences of two leaf-cutter ants, *Acromyrmex echinatior* and *Atta cephalotes* (385 and 376 putative functional genes, respectively). These were used, together with *Or* genes from *Camponotus floridanus*, *Harpegnathos saltator*, *Pogonomyrmex barbatus*, *Linepithema humile*, *Cerapachys biroi*, *Solenopsis invicta* and *Apis mellifera*, in molecular evolution analysis. Like the *Or* family in other insects, ant *Or* genes evolve by the birth-and-death model of gene family evolution. Large gene family expansions involving tandem gene duplications, and gene gains outnumbering losses, are observed. Codon analysis of genes in lineage-specific expansion clades revealed signatures of positive selection on the candidate cuticular hydrocarbon receptor genes (9-exon subfamily) of *Cerapachys biroi*, *Camponotus floridanus*, *Acromyrmex echinatior* and *Atta cephalotes*. Positively selected amino acid positions are primarily in transmembrane domains 3 and 6, which are hypothesized to contribute to the odor-binding pocket, presumably mediating changing ligand specificity.

**Conclusions:**

This study provides support for the hypothesis that some ant lineage-specific *Or* genes have evolved under positive selection. Newly duplicated genes particularly in the candidate cuticular hydrocarbon receptor clade that have evolved under positive selection may contribute to the highly sophisticated lineage-specific chemical communication in each ant species.

**Electronic supplementary material:**

The online version of this article (doi:10.1186/s13104-015-1371-x) contains supplementary material, which is available to authorized users.

## Background

Ants are a very successful group of insects accounting for more than 11,800 species [1]. They exploit a wide range of terrestrial habitats and have important roles in ecosystems including herbivore, predator, symbiotic interactions and nutrient cycling [2]. Ants are eusocial insects with well-organized and regulated societies. Each individual has roles responsible for the survival of their colony. Social behaviors of many ants have long fascinated people, including the leaf-cutter ants that forage for fresh leaves and use them as materials for their fungus farming, or slave-making ants that capture brood from other ant species and enslave them as workers [1].

Chemical communication is presumably the most important method in ant communication as multiple ant pheromones have been identified, e.g. trail, aggregation, alarm, territory and sex pheromones [3]. In addition, cuticular hydrocarbons play important roles in nest-mate recognition, dominance and fertility cues, task decision making and chemical mimicry [4–7]. Thus, the ant chemosensory system must have evolved to be able to perceive these numerous chemicals, many of which differ by species.

Our understanding of the molecular basis and neuronal circuitry of the insect olfactory system has improved greatly during the past decade [8, 9]. Insect odorant receptors (*Ors*) are heterodimers of a specific ligand-binding receptor and a conserved co-receptor (Orco) forming a ligand-gated ion channel [10–13]. Many ligand-receptor relationships have been identified which illuminate insect behavioral responses to odorant cues e.g. [14–17]. The related gustatory receptor family and the unrelated ionotropic receptor family also play roles in chemoreception, both for taste and smell [18, 19], however the scope of this work is limited to investigation of the odorant receptor gene family.

Recent advances in genomic studies made it possible to identify the whole *Or* gene repertoire from many ant species including the Florida carpenter ant *Camponotus floridanus* (352 *Ors*) and the Jerdon’s jumping ant *Harpegnathos saltator* (347 *Ors*) [13, 20], the red harvester ant *Pogonomyrmex barbatus* (344 *Ors*) and the Argentine ant *Linepithema humile* (337 *Ors*) [21, 22] and recently the clonal raider ant *Cerapachys biroi* (368 *Ors*) [23], as well as a partial set of *Ors* in the red imported fire ant *Solenopsis invicta* (259 *Ors*) [24]. In addition, *Or* genes have been identified from two related hymenoptera, the honey bee *Apis mellifera* (164 *Ors*) [25] and the jewel wasp *Nasonia vitripennis* (225 *Ors*) [26]. These provide a comparative platform for molecular evolution analysis, but herein we only utilize the honey bee as it is more closely related to the ants. Understanding evolutionary changes in the ant *Or* genes may shed light on the evolution of social communication in ants. Draft genome sequences of two leaf-cutter ants, *Acromyrmex echinatior* and *Atta cephalotes*, are also available [27, 28], but their complete *Or* gene repertoires have not been reported, indeed their automated annotation pipelines only modeled 8 and 30 *Or* genes, respectively. Thus, the first aim of this study was to identify *Or* genes in the *Ac. echinatior* and *At. cephalotes* genomes to be used in molecular evolution analysis of the ant *Or* gene family. The second aim was to test whether and how positive selection might have operated in the ant *Or* gene family. We hypothesized that *Or* genes that are different between ant species, e.g. lineage-specific expansion genes, might be associated with olfactory adaptation unique to each ant species through diversification to recognize different species-specific ligands. If the amino acids involved in this diversification are in the same locations in the proteins, we predicted that signals of positive selection should be found for these *Or* genes and specific amino acid positions.

## Results

### The *Ac. echinatior* and *At. cephalotes* odorant receptor genes

We manually annotated 435 candidate *Or* genes from the *Ac. echinatior* genome. Among these, 385 genes (89 %) are intact gene models (306 full and 79 partial gene models) and 50 genes (11 %) are putative pseudogenes. For *At. cephalotes*, there are 434 candidate *Or* genes; 376 intact genes (87 %) (281 full and 95 partial gene models) and 58 (13 %) putative pseudogenes (Additional file 1). We note that the ant *Or* gene models from subfamily Q-U are still incomplete (possibly missing one short exon at the N-terminus) and require experimental evidence to extend these gene models. This leads to the missing N-terminus in the gene models from subfamily Q-U of the two leaf-cutter ants (*AecOr183*-*AecOr231* and *AceOr184*-*AceOr230*). The average length of OR proteins encoded from intact gene models was 392 amino acids for both species.

**Fig. 1:**
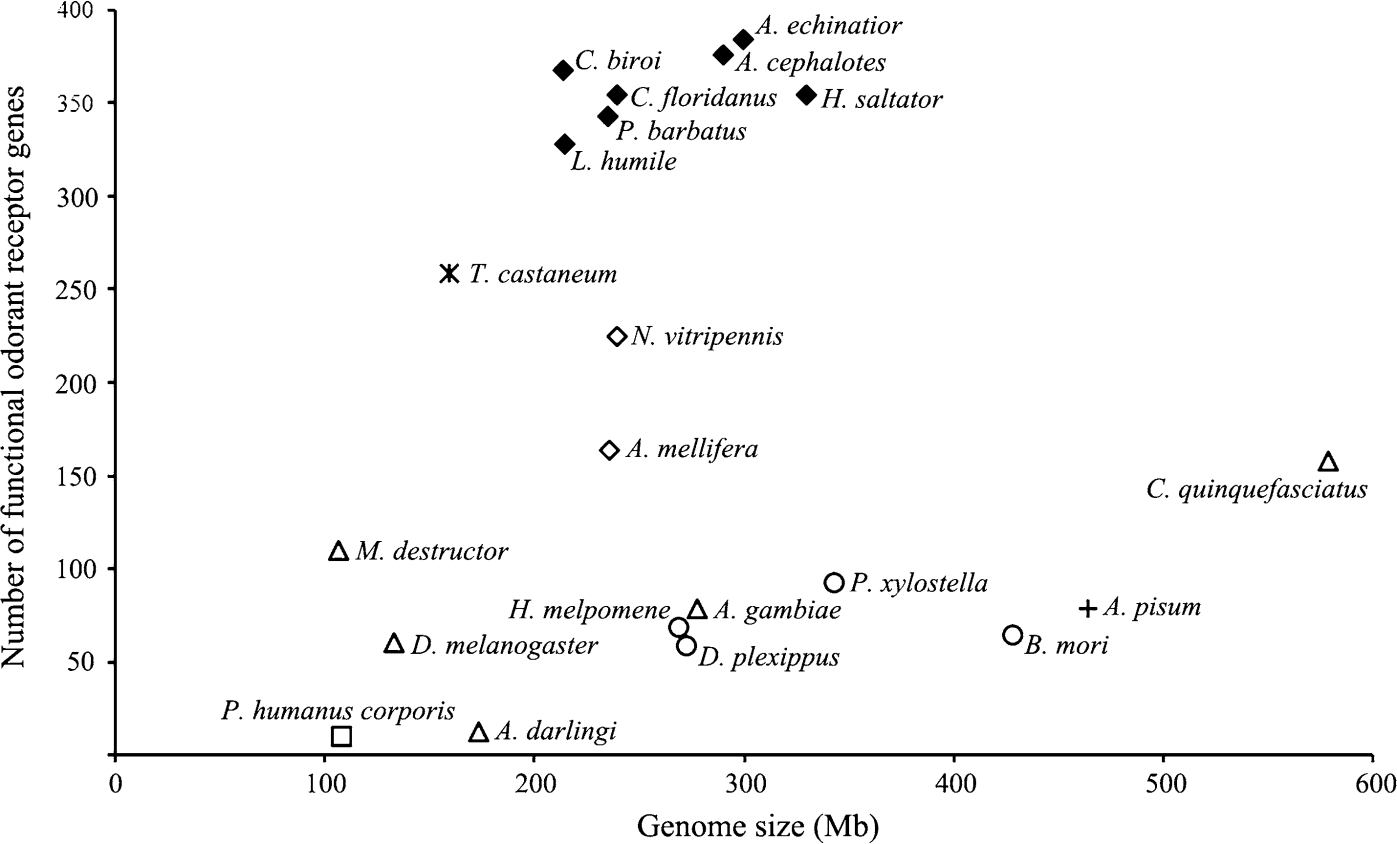
The number of functional *Or* genes vs. genome size in different insects. *Triangle* Diptera; *circle* Lepidoptera; *open diamond*/*closed diamond* Hymenoptera/ants; *asterisk* Coleoptera; *plus symbol* Hemiptera; *open square* Phthiraptera. Reference: Phthiraptera: [74]; Diptera: [32, 35, 75–80]; Hemiptera: [51, 81]; Lepidoptera: [30, 31, 82–85]; Hymenoptera: [20–28, 86, 87]; Coleoptera: [88, 89]

The number of putative functional *Or* genes in *Ac. echinatior* and *At. cephalotes* is the highest number reported so far in insects, even slightly higher than the other available ants (328 - 368 genes) [23]. Other hymenopteran insects also have high numbers of *Or* genes e.g. 301 *Ors* in *N. vitripennis* [26] and 157 *Ors* in *Ap. mellifera* [25]. Insects from other groups have much lower numbers of *Or* genes e.g. 59–93 *Ors* in Lepidoptera [29–31] and 63-158 *Ors* in Diptera [32–35]. We asked whether this variation in number of *Or* genes can be partly explained by the size of insect genomes. In Fig. 1, we compare number of *Or* genes against the genome sizes. There is no significant association between insect genome size and number of *Ors* (*r* = 0.003, *P* = 0.99) or ant genome size and number of ant *Ors* (*r* = 0.51, *P* = 0.25), thus *Or* gene family size is presumably primarily related to the ecological requirements of each species.

Most of the *Ac. echinatior* and *At. cephalotesOr* genes are found in clusters on particular scaffolds in the genome assembly. Only 25 genes (6 %) in *Ac. echinatior* and 28 genes (6 %) in *At. cephalotes* are found as singletons. Some clusters are greatly expanded, for example, 49 *Ors* (*AceOr282*-*AceOr330*) spanning ~450 kb of Scaffold 28, 49 *Ors* (*AecOr288*- *AecOr336*) spanning ~320 kb of scaffold 507, 56 *Ors* (*AecOr232*-*AecOr287*) spanning ~400 kb of scaffold 7, 59 *Ors* (*AecOr84*-*AecOr142*) spanning ~270 kb of scaffold 397, and 79 *Ors* (*AceOr81*-*AceOr159*) spanning ~440 kb of Scaffold 5. The mean number of genes per cluster is 11.0 ± 2.4 genes for *Ac. echinatior* and 13.7 ± 3.5 for *At. cephalotes*. There is no difference in mean cluster size between *At. cephalotes* and *Ac. echinatior* (unpaired *t* test, *P* = 0.53).

## Phylogenetic relationships

**Fig. 2:**
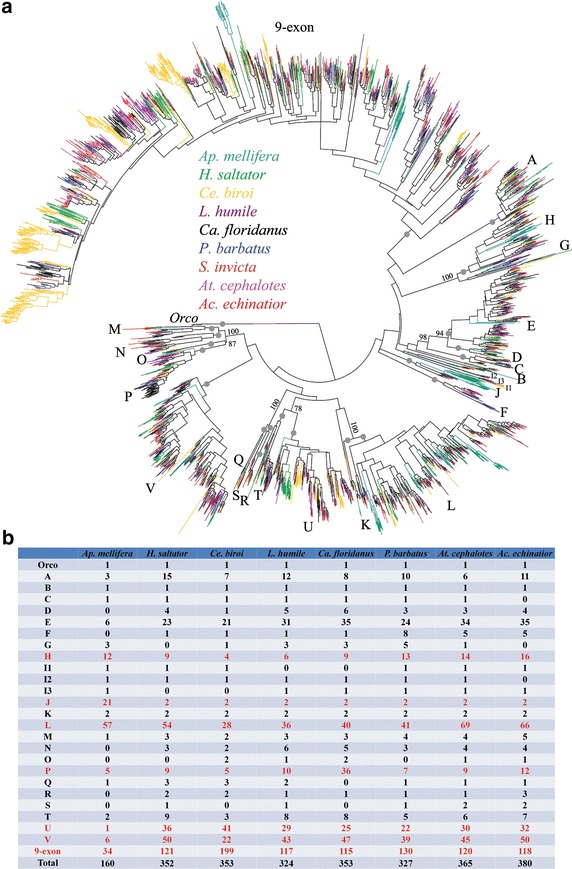
Phylogenetic relationship of Hymenoptera *Or* genes. **a** A maximum-likelihood tree of the 3330 *Or* genes from 9 hymenopteran species. Tree was made using RAxML under the model JTT and GAMMA correction. Node reliability was evaluated by 100 bootstrap replications. *Grey dots* indicate bootstrap values (>80) of each *Or* subfamily. Bootstrap values for nodes representing relationships among subfamilies are shown only if the value is higher than 70. Branches are colored according to species (*Apis mellifera*,* light blue*; *Harpegnathos saltator*,* green*; *Cerapachys biroi*,* yellow*; *Linepithema humile*,* purple*; *Camponotus floridanus*,* black*; *Pogonomyrmex barbatus*,* blue*; *Solenopsis invicta*,* brown*; *Atta cephalotes*,* pink*; *Acromyrmex echinatior*,* red*). A full tree with gene names and branch support is provided in Additional file 2. **b** Comparison of number of *Or* genes (only putative functional proteins) in each subfamily. Subfamilies that underwent sub-lineage expansion/contraction are highlighted in *red*

Our phylogenetic tree supports monophyletic status of previously identified ant *Or* subfamilies (24 subfamilies: Orco, A–V, and the large 9-exon subfamily) (Fig. 2; Additional file 2) [13, 21, 23]. However, we propose that subfamily I should be divided into I1, I2 and I3 because they have different gene models (7-exons|002020, 8-exons|2200200, and 5-exons|2000, respectively, where the intron phases are shown after the pipe) (diagrams of gene models for each ant *Or* subfamilies are provided in Additional file 3). Furthermore, phylogenetic relationships suggest a simple 1:1 ratio for orthologous genes from the 8 ant species in the I2 and I3 subfamilies, but not subfamily I1 (Additional file 2). Branch support for each subfamily is higher than 80 but support between subfamilies is usually low. Only a few sister group relationships between subfamilies are identified, e.g. subfamily M-P (100 % bootstrap support), Q + R (100 %), E + D (94 %), (E + D) + C (98 %), and T + U (78 %), suggesting that most subfamilies have been diverging for a long time.

Many cases of lineage-specific expansion were observed, especially for the large 9-exon subfamily. These include a large expansion of >60 *CbirOr* genes (clade Cbir6), >50 *CbirOr* genes (clade Cbir4 + 5), and >40 *CbirOr* genes (clade Cbir6). The largest lineage-specific expansion in other subfamilies is >30 *HsOr* genes (U-Hsal1) (Additional file 2).

For the two relatively closely-related leaf-cutter ants, their *Or* genes are very similar. Orthologous relationships between their *Ors* are observed along the tree, as expected because their divergence time is relatively recent (~8 MY ago). We also observed large gene expansions specific to the leaf-cutter ant lineage (clade $1 in 9-exon3 and 9-exon4) (Additional file 2). Our phylogenetic tree shows that genes that are located in the same scaffold cluster are closely related suggesting that they arose via tandem gene duplication resulting from unequal crossing over.

The number of genes in each subfamily varies greatly from 1 to 199 genes (Fig. 2b). As reported previously for ants [13, 21–24], the 9-exon subfamily is the largest, accounting for ~38 % of the ant *Or* gene family (an average percentage of the 7 ant species), with a maximum of 199/353 in *Ce. biroi* (~56 %). The ants generally have twice as many *Or* genes as the bee. We tested whether genes in each subfamily deviated from this ratio, which may indicate independent gene expansion/contraction in some species. We found 8 subfamilies (F, H, J, L, P, U, V, 9-exon) where their number of genes deviated from this ratio (*χ*^2^ test, *P* < 0.05).

### Gene gains and losses

**Fig. 3:**
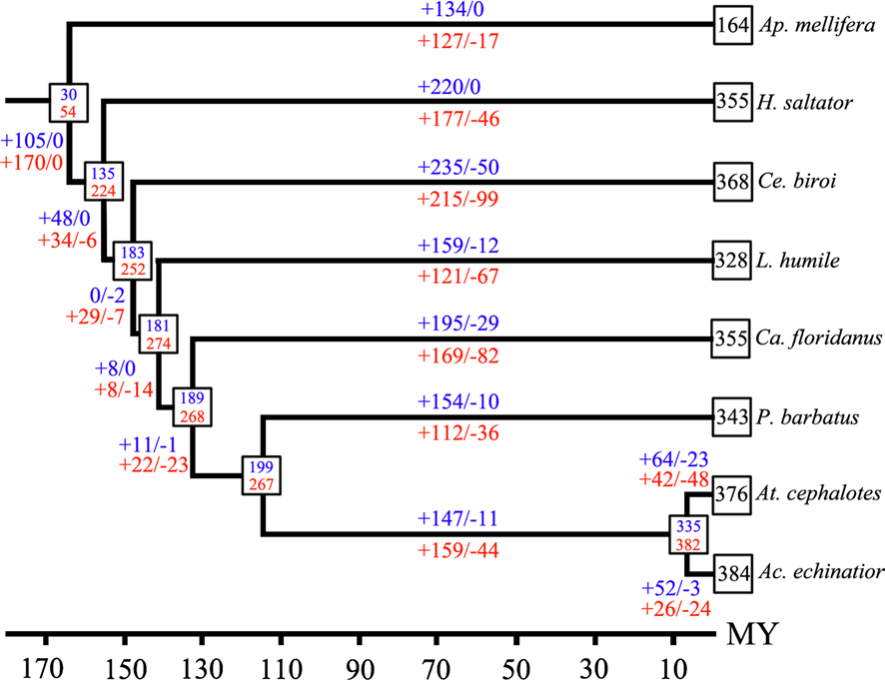
Estimation of gene gain and loss events and number of *Or* genes in ancestral species. Number of gene gains and losses are shown on tree branches and indicated with+ and −, respectively (*blue* BadiRate, *red* NOTUNG). *Numbers in square boxes* indicate number of *Or* genes in ancestral and extant species. The phylogeny of 7 ants and a honey bee (*Ap. mellifera*) with approximate divergence times (in million years: MY) is inferred from references [2, 62, 63]

Two methods used for the analysis reveal similar trends in the gene family evolution but estimate different numbers of ancestral gene copies and numbers of gene gain and loss events. According to the maximum-likelihood analysis implemented in BadiRate, the estimated number of *Or* genes in the most recent common ancestor of bees and ants was 30 genes (Fig. 3). The number increased to 135 genes (>4 fold) in the common ancestor of the seven ants after the separation of bee and ant lineages. The number of *Or* genes in the ant ancestors living around 155–115 MY ago did not change much (135–199 *Or* genes) with approximately 67 gene gains and 3 gene losses in total. However, extensive gains and considerable losses are inferred on lineages leading to extant ant species. During the past 155 MY from the most recent common ancestor of these 7 ants, the number of *Or* genes increased significantly to around 328–384 genes.

On the other hand, NOTUNG estimates higher numbers of ancestral gene copies. The common ancestor of bees and ants was estimated to have 54 *Or* genes. Similar to the results of BadiRate, the number increased around 4 fold in the common ancestor of the seven ants (224 genes). Extensive gene gains outnumbering losses were also inferred on lineages leading to extant ant species.

The common ancestor of *At. cephalotes* and *Ac. echinatior* living around 8 MY ago was estimated to have 335 genes by BadiRate and 382 genes by NOTUNG. BadiRate suggests that gene gains outnumbered gene losses leading to larger numbers of *Or* genes in the two leaf-cutter ants. On the contrary, NOTUNG suggests that both gene gains and losses occurred evenly leading to approximately the same number of *Or* genes in the extant ant species.

### Pattern of positive selection

To examine the prediction of positive selection on the newly duplicated *Or* genes, we used PAML to perform site tests on 31 lineage-specific expansion *Or* clades (Table 1 and Additional file 2) (*Ap. mellifera*—9 clades, *Ce. biroi*—8 clades, *Ca. floridanus*—6 clades, *H. saltator*—5 clades, *P. barbatus*—2 clades, *L. humile*—1 clade), 15 of which are clades in the 9-exon *Or* gene subfamily. All of these clades have low mean *d*_*S*_ values (*d*_*S*_ < 1) suggesting that their sequences are not too divergent and thus should not suffer from saturation of synonymous changes, which are known to cause false positive detection in CodeML [36, 37].

**Table 1:** Site tests for positive selection on lineage-specific expansion *Or* clades

Clade	n^a^	d_S_^b^	2Δl^c^			M1a vs M2a *P* value (with Bonferroni correction)
			M7 versus M8 (df = 2)	M8a versus M8 (df = 1)	M1a versus M2a (df = 2)	
9-exon subfamily						
9-exon Amor1	8	0.40	0.14	0.03	0	1.0
9-exon Amor2	8	0.54	7.74*	0.08	0	1.0
9-exon Amor3	13	0.10	7.21*	5.34*	0	1.0
9-exon Cbir1	13	0.13	20.72**	5.01*	4.27	1.0
9-exon Cbir2	19	0.12	75.00**	54.45**	62.29**	9.22E-13**
9-exon Cbir3	9	0.16	16.82**	7.85*	7.79*	0.6290
9-exon Cbir4	13	0.07	51.78**	47.63**	47.05**	1.88E-09**
9-exon Cbir5	16	0.07	98.64**	83.20**	84.04**	0**
9-exon Cbir6	25	0.07	105.71**	98.16**	106.90**	0**
9-exon Cfor1	6	0.19	29.77**	17.19**	14.61**	0.0021*
9-exon Cfor2	7	0.14	43.76**	38.44**	37.27**	2.50E-07**
9-exon Cfor3	7	0.20	8.19*	4.95*	0	1.0
9-exon Hsal1	7	0.27	2.67	0	0	1.0
9-exon Hsal2	7	0.14	15.09**	9.93*	12.33*	0.0652
9-exon Pbar1	9	0.10	8.62*	8.62*	0	1.0
Other subfamilies						
H-Amor1	11	0.39	12.55*	2.32	0	1.0
J-Amor1	19	0.35	17.60**	0.42	0	1.0
L-Amor1	14	0.23	8.79*	7.82*	0	1.0
L-Amor2	7	0.23	3.04	2.08	0	1.0
L-Amor3	8	0.31	1.60	0.48	0	1.0
L-Amor4	20	0.20	3.33	0.41	0	1.0
U-Cbir1	7	0.11	2.10	0.00	0	1.0
U-Cbir2	6	0.11	6.66*	5.98*	5.19	1.0
P-Cflo1	31	0.11	30.31**	9.63*	0	1.0
V-Cflo1	11	0.11	1.02	0.78	0	1.0
V-Cflo2	7	0.12	14.23**	2.17	2.03	1.0
E-Hsal1	8	0.18	0.00	0.00	0	1.0
U-Hsal1	33	0.11	8.72*	0.13	0	1.0
V-Hsal1	10	0.06	1.09	0.70	0	1.0
P-Lhum1	7	0.20	6.69*	2.94	0	1.0
F-Pbar1	6	0.12	1.55	1.30	0.46	1.0

^a^Number of sequences in the data set

^b^Mean d_S_ for all branches estimated under M0 model

^c^Log Likelihood ratios; 2x(lnL_H1_ − lnL_H0_)

** Significant at the 0.1 % level

* Significant at the 5 % level

We detected signatures of positive selection in 21 *Or* gene clades when we tested model M8 (beta&ω) against M7 (beta) (12 of them were significant at the 0.1 % level). However, the number of clades with signatures of positive selection was reduced to 15 and 8 under the more stringent tests of M8 (beta&ω >1) vs. M8a (beta&ω) and M2a (selection) vs. M1a (neutral), respectively. To avoid false positive results, we focus only on clades that had significant results from the most stringent test, M1a vs. M2a. After Bonferroni correction, to reduce false-positive detection due to multiple tests, positive selection was confidently detected in 5 ant *Or* clades (P < 0.001), 4 clades from *Ce. biroi* and 1 clade from *Ca. floridanus*. All of them are from the 9-exon subfamily.

We then used the branch-site test [38] to examine if positive selection operated on duplicated *Or* genes that only expanded in specific ant lineages. We conducted the test on 10 *Or* gene clades (Table 2). After Bonferroni correction, 2 clades (9-exon2 $1 Aech & Acep and 9-exon3 $1 Aech & Acep) exhibited positive selection (*P* < 0.001), and again they are from the 9-exon subfamily, and both are leaf-cutter ant lineage-specific clades.

**Table 2:** Branch-site tests for positive selection on lineage-specific expansion foreground branches

Clades	n^a^	d_S_^b^	lnL		2Δl^c^ (df = 1)	*P* value (without Bonferroni Correction)	*P* value (with Bonferroni Correction)
			Null model	Alternative model			
9-exon1 $1 Aech & Acep	11 (6)	0.17	−7178.30	−7177.54	1.52	0.218	3.045
9-exon2 $1 Aech & Acep	20 (6)	0.11	−10419.64	−10408.81	21.65	3.27E-6**	4.57E-5**
$2 Pbar	20 (5)	0.11	−10426.11	−10422.62	6.98	8.26E-3*	0.116
9-exon3 $1 Aech & Acep	34 (32)	0.07	−12015.98	−11995.80	40.36	2.11E-10**	2.96E-09**
9-exon4 $1 Aech & Acep	23 (14)	0.11	−10103.70	−10099.98	7.43	6.42E-3*	0.089
A1 $1 Hsal	10 (5)	0.21	−8010.95	−8010.95	3.95	1	1
H1 $1 Aech & Acep	8 (6)	0.12	−3976.99	−3976.99	0.00	1	1
L1 $1 Aech & Acep	15 (7)	0.16	−8415.00	−8415.00	0.00	1	1
L2 $1 Aech & Acep	11 (9)	0.12	−5629.05	−5629.05	0.00	1	1
L3 $1 Aech & Acep	24 (20)	0.10	−11115.08	−11115.08	0.00	1	1
U1 $1 Aech & Acep	43 (16)	0.13	−22254.33	−22254.33	0.00	1	1
$2 Cflo	43 (6)	0.17	−22277.77	−22277.77	0.64	1	1
$3 Lhum	43 (10)	0.11	−22256.65	−22256.33	1.52	0.4233	1

^a^Number of sequences in the data set with number of sequences in the foreground branch in the parentheses

^b^Mean d_S_ for all branches estimated under M0 model

^c^Log Likelihood ratios; 2x(lnL_H1_ − lnL_H0_)

** Significant at the 0.1 % level

* Significant at the 5 % level

**Fig. 4:**
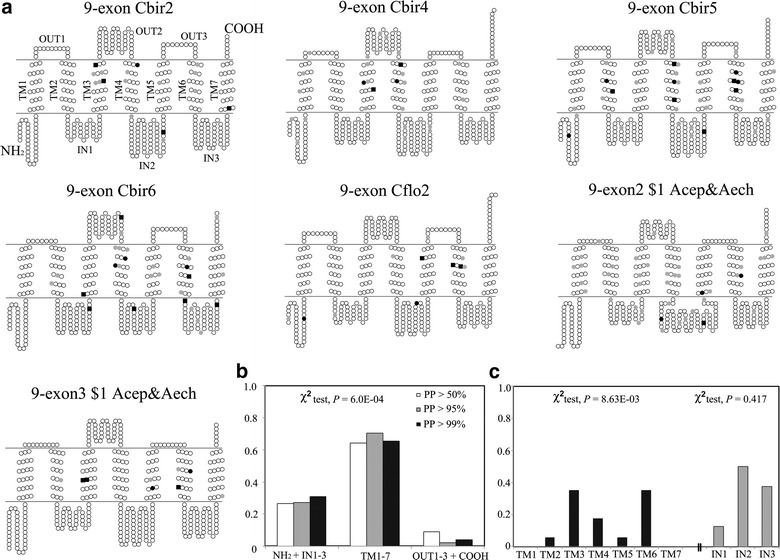
Distribution of positively selected sites on the ant odorant receptor proteins. **a** Each protein schematic represents an odorant receptor from gene clades that show signatures of positive selection (Tables 1, 2). Protein topology was predicted using TOPCON, TMHMM and TOPPRED. These proteins have 7 transmembrane domains with an intracellular N terminus and extracellular C terminus (*TM* transmembrane membrane region, *IN* intracellular loop, *OUT* extra cellular loop). Each *circle* indicates an amino acid. Positively selected sites (PSSs) predicted under model M2a are highlighted as follow: *grey circle* PSSs with posterior probability (PPs) > 50 %, *black circle* PSSs with PPs > 95 %, *black square* PSSs with PPs > 99 %. **b** Proportion of positively selected sites in each protein region. **c** Proportion of PSSs in each TM region (*left*) and proportion of PSSs in each intracellular loop (*right*)

We further examined the distribution of positively-selected sites (PSSs) as inferred from model M2a (site test) and alternative model (branch-site test) on the receptor protein topology. The PSSs (PPs > 50 %) are not randomly distributed in different regions of the proteins (N-terminus and intracellular loops, transmembrane regions, extracellular loops and C-terminus) (Fig. 4a) (χ^2^ test, *P* = 0). The same observation was made when only sites with PPs > 95 % (*P* = 1.0E-4) and > 99 % (*P* = 6.0E-04) were considered (Fig. 4b). The proportion of positively-selected sites in each protein region was highest in the transmembrane regions follow by intracellular loops. We further tested the distribution pattern of positively-selected sites in different TM regions (TM1-TM7), and they are not distributed randomly in different transmembrane regions (χ^2^ test, *P* = 8.63E − 03). PSSs in TM3 and TM6 accounted for 70 % of the PSSs located in TM regions. However, there is no difference in the proportion of PSSs in the intracellular loops 1, 2 and 3 (χ^2^ test, *P* = 0.417).

## Discussion

### Expanded ant odorant receptor gene family

It has been well documented that the number of *Or* genes in ant genomes is much larger than those of most other insect groups [13, 21, 23] (Fig. 1). We identified a large number of *Or* genes from two leaf-cutter ant genomes, *Ac. echinatior* and *At. cephalotes* (385 and 376 putative functional genes, respectively), supporting these previous observations. The larger number of *Or* genes may lead to a more complex sensory processing in the ant brain [39]. Indeed, elaboration of the ant olfactory system is also present at the anatomical level as they have a large number of glomeruli (the first olfactory processing unit) in the antennal lobe of the brain. A previous study reported that the number of glomeruli in 7 *Atta* spp. varies from 336 to 452 while the number in 7 *Acromyrmex* spp. varies from 369 to 477 [40]. A large number of odorant receptors and glomeruli could provide numerous and complex combinatorial olfactory codes that might allow ants to detect and discriminate a wide range of odorant stimuli that are important for their ecological success.

### Birth and death evolution

The insect odorant receptor gene family evolves under the birth-and-death mode of evolution as evident from repeated gene duplication, pseudogenization, and loss [41, 42]. Our analysis in the ant *Or* gene family strongly supports this model. In addition, *Ac. echinatior* and *At. cephalotesOr* genes are mostly found in clusters on DNA scaffolds and some clusters are greatly expanded. Phylogenetic analysis suggests that these clustered genes arose from tandem gene duplication as genes in the same cluster shared close relationships. A large number of pseudogenes are also observed in the *Ac. echinatior* (11 %) and *At. cephalotes* (13 %) *Or* gene family, which is the first step towards gene loss.

Our estimates of gene gain and loss using BadiRate and NOTUNG [43–45] suggest highly dynamic changes in the number of *Or* genes over the evolution of ants (Fig. 3). In this study, NOTUNG suggests that the numbers of *Or* genes in the common ancestor of ants and bee and in the common ancestor of ants were 54 and 224, respectively. These numbers are similar to those estimated by NOTUNG in the previous study which used fewer ant species (51 and 204 *Or* genes, respectively) [13] suggesting the consistency of this method. Our maximum-likelihood estimation using BadiRate suggests lower numbers for these ancestors (30 and 135 genes, respectively). The differences could be explained by the fact that we used OrthoMCL to define orthologous groups of the *Or* genes. This method strictly identifies orthologous relationships based on reciprocal best hit using BLAST which in turn limits the number of genes shared in all species, leading to a small number of *Or* genes estimated in the common ancestor. Nevertheless, these methods suggest similar trends in ant *Or* gene evolution, that is, a large number of gene gains over time especially on the lineage leading to the extant species and a lower but considerable number of gene losses. That such gene family expansions reflect relevant ecological features of animals is generally established, for example, ecologically-relevant gene families commonly co-vary in size across species e.g. [46].

Phylogenetic analysis assigned ant *Or* genes into 26 well-supported subfamilies (Fig. 2a, b). The number of genes in each subfamily varies greatly. Some subfamilies have only a few genes and the ratio of number of genes between species is roughly 1:1 e.g. subfamilies Orco, B, C, I1, I2, I3 and K. Genes in these clades may have conserved and particularly important roles because they were retained in the genome since the common ancestor of bees and ants. A conserved role for *Orco* as a partner protein in ion channel signaling is well known [10–13]. In contrast, genes in some subfamilies underwent substantial expansion, e.g. subfamilies J, L, U, V and 9-exon. This suggests that gene gains and losses did not occur randomly. Particular subfamilies retain their size while other subfamilies experience gene gains and losses. Unfortunately, our very limited knowledge of ligand-receptor specificity makes it impossible to understand why each subfamily evolved different sizes.

### 9-exon subfamily

The most striking feature of the ant *Or* gene evolution is the dramatic expansion of the 9-exon subfamily. The number of genes in the 9-exon subfamily accounted for more than a third of the entire family, for example, in *Ce. biroi* the 9-exon subfamily is about six fold larger than in *Ap. mellifera*. *Or* genes from the 9-exon subfamily were previously proposed to be candidates for cuticular hydrocarbon (CHC) receptors in ants [21, 22]. CHCs are long non-volatile chemicals of enormous variety (>100 chemicals) found in ant cuticle that have important roles in nest-mate, developmental stage, caste, and species recognition [47–49]. Due to the diversity of ant CHCs, their receptors should also be as diverse. It seems that only the 9-exon subfamily is large enough to perform the task. This hypothesis was later supported by gene expression studies in *Ca. floridanus* and *H. saltator* where most genes in the 9-exon subfamily were expressed at higher level in the female workers, which are mostly responsible for kin recognition [1], compared to the male drone [13]. A subset of 9-exon genes, however, are highly enriched in males, raising the possibility that they have roles in detecting queen mating pheromones.

### Darwinian positive selection

From the total of 41 *Or* gene clades tested, signatures of positive selection were detected in 7 ant lineage-specific expansion *Or* clades; four clades from *Ce. biroi* (9-exon Cbir2, 9-exon Cbir4, 9-exon Cbir5, 9-exon Cbir6), one clade from *Ca. floridanus* (9-exon Cfor2), and two clades from leaf-cutter ant lineages (9-exon2 $1 Aech & Acep and 9-exon3 $1 Aech & Acep) (Tables 1, 2). This finding provides strong support that Darwinian positive selection has had an important role in shaping evolution of the newly duplicated *Or* genes resulting in olfactory adaptation unique to each ant lineage.

All clades that show signatures of positive selection are from the 9-exon subfamily. As discussed above, most genes in this subfamily may function in CHC reception in female workers and a few of them may function as queen pheromone receptors in drones. The clonal raider ant, *Ce. biroi*, is a subterranean ant lacking developed eyes. It is assumed that this species requires a better sense of chemoreception for social communication to compensate for the loss of sight and for specialization in recognizing pheromones of other ant species [23], which might explain the great expansion of the 9-exon subfamily in *Ce. biroi* and positive selection that is detected on four clades of *CbirOr* genes.

Although positive selection was detected only on 9-exon subfamily clades, we do not exclude the possibility that positive selection also operated on other subfamilies but the method we used might not be able to detect it. A previous study investigated signatures of positive selection in 7 ant genomes using branch-site models on all branches of a limited hymenopteran OR tree (*P. barbatus*, *L. humile* and *N. vitripennis*) [50]. Twenty three percent of branches tested displayed significant signals for positive selection. Interestingly, positive selection was detected more on branches leading to the solitary wasp species compared to social ants (40 vs. 19 %). Their result challenges the hypothesis that genes involved in chemical signaling experienced increased positive selection in social insects, however their analyses were done on all clades of *Or* genes, some of which may not be responsible for the detection of odorant ligands involved in social communication, thus the differences in number of branches that show positive selection may not directly represent selection in the *Or* genes involved in social interaction. Positive selection that we detected in genes in the 9-exon subfamily does support the hypothesis that *Or* genes involved in social communication experienced increased positive selection in social insects.

The proportion of positively selected sites (codons) was highest in the TM regions especially TM3 and TM6 (Fig. 4). These sites may play roles in ligand-binding specificity of the ant ORs. A previous positive selection study of *Or* paralogs in the pea aphid also showed the highest proportion of PSSs in the TM regions [51]. Important roles of amino acids in the transmembrane regions in ligand-binding specificity have been demonstrated in heterologous cell expression systems, e.g. amino acids in TM3 of *Drosophila* OR85b detecting 2-heptanone [52], TM2 of *Drosophila* OR59b detecting 1-octen-3-ol [53], TM3 of OR3 in *Ostrinia* moths detecting female sex pheromone [54], and EC2 and TM4 of AgamOR15 in *Anopheles gambiae* mosquitoes and multiple odorants [55]. Unfortunately our knowledge of ligand-receptor specificity in ant odorant receptor is still limited, with only two ligand-receptor relationships known, *Ca. floridanus* CfOR263 and 2,4,5-trimethylthiazole and *H. saltator* HsOR55 and 4-methoxyphenylacetone [13]. Comparable experimental work will be required to confirm whether the predicted positively selected sites are involved in ligand-binding specificity of the ant cuticular hydrocarbons.

## Conclusions

We describe the *Or* gene family from two leaf-cutter ants, *Acromyrmex echinatior* and *Atta cephalotes* (385 and 376 putative functional genes, respectively). Molecular evolution analyses on the ant *Or* genes revealed processes similar to those reported for other insect *Or* genes. These include large lineage-specific gene subfamily expansions by means of tandem gene duplication, birth and death of genes, and dynamic changes of gene gain and loss events during the evolution of ants. Using codon analysis, we detected signatures of positive selection on the lineage-specific expansion *Or* gene clades from the 9-exon subfamily, which are candidates for cuticular hydrocarbon receptors in ants [21, 22]. This study supports the hypothesis that highly specialized olfactory sense in ants, in this case lineage-specific chemical communication evolved under positive selection.

## Methods

### Identification of the *At. cephalotes* and *Ac. echinatior* Odorant Receptor Genes

We identified the remaining *Or* family members in these two genomes following the partially automated approach of Ref. [13]. Briefly, the amino acid sequences of the odorant receptors (ORs) from *P. barbatus*, *S. invicta*, *Ca. floridanus*, *L. humile* and *Ce. biroi* were used as queries for tBLASTn searches against the *At. cephalotes* and *Ac. echinatior* genome databases at the Ant Genomes Portal (Acep_1.0 and Aech_V2.0 Scaffold assembly, respectively; http://hymenopteragenome.org/ant_genomes) [56]. Search parameters were set as default except that Expect threshold (E value) was changed to 1000 to allow the detection of highly divergent sequences. Blast results were used to build draft gene models in a text editor. The DNA sequences of putative gene regions were retrieved using the GBrowse tool and compared with ORs that are closely related using GeneWise [57]. GeneWise allows the prediction of coding proteins based on similarity of translated DNA and input protein sequence. Putative ORs inferred from GeneWise were aligned with known ant ORs, and problem regions of models and pseudogenes were refined. We performed the BLAST and following annotation steps for multiple iterations until no new genes were discovered.

Gaps in the genome assemblies prevent the building of some full-length gene models. We only kept gene models that encode more than 250 amino acids (i.e. more than 60 % of the average full-length ant OR protein) in our final gene set. We added suffices after gene names to indicate incomplete gene models in a similar way to previous studies [21–23] as follow: NTE = N terminus missing, CTE = C terminus missing, I = internal sequence missing. If more than one region was missing, the first syllabus (N/C/I) was used. We identified many pseudogenes based on premature stop codons, frameshifts and incorrect splice sites. We used suffix “PSE” (or “P” with other suffixes) to indicate pseudogenization. Given the possibility that some of these putative pseudogenes were incorrectly labeled as such due to potential errors in the genome assembly, we added suffix “(S)” for possible non-canonical splice site and “(F)” for possible false frameshift.

### Phylogenetic analysis

We constructed a phylogenetic tree of *Or* genes from 9 hymenopteran insects (8 ants and 1 bee) including *At. cephalotes*, *Ac. echinatior*, *P. barbatus*, *L. humile*, *S. invicta*, *H. saltator*, *Ca. floridanus*, *Ce. biroi* and *Ap. mellifera* [13, 21–25]. Only genes encoding proteins longer than 250 amino acids were included in the analysis. Amino acid sequences were aligned using MUSCLE v3.8.31 [58] followed by manual adjustment using BioEdit (http://www.mbio.ncsu.edu/bioedit/bioedit.html). Poorly aligned regions (sites contain more than 30 % gaps) in the alignment were discarded using TrimAl v1.2 [59]. The final alignment contains 3330 OR proteins and 412 characters. Phylogenetic analysis was performed using RAxMLv8.0 [60] under the model JTT and GAMMA correction with 100 bootstrap iterations. The phylogenetic tree was displayed and labeled using FigTree v1.4 (http://tree.bio.ed.ac.uk/software/figtree/). A full tree with clade labels is provided in the Additional file 2.

### Estimation of gene gain and loss events

We used the maximum-likelihood approach implemented in BadiRate v1.35 [43] to estimate the number of *Or* gene gain and loss events during the evolution of these hymenopteran insects (excluding *S. invicta* due to its incomplete gene dataset). In brief, we first inferred orthologous groups of these *Or* genes based on reciprocal best hits within and between gene families of each hymenopteran species using the OrthoMCL software (inflation of 1.5 and e value threshold of 10^−5^) [61]. We then inferred the phylogenetic tree of these hymenopteran species with branch lengths reflecting divergence times from previous phylogenetic studies [21, 62, 63]. For each orthologous group, gene gain and loss events were counted from the number of members at internal nodes inferred by maximum likelihood under the BDI stochastic model [64], assuming that each branch has its own specific turnover rates (BDI-FR-CML method) [65]. As an alternative approach, we used a tree reconciliation method implemented in NOTUNG 2.6 to conduct the test [44, 45]. In brief, we constructed a phylogenetic tree of *Or* genes (the same dataset used in the BadiRate analysis) using FastTree 2.1 with default settings [66]. NOTUNG reconciled gene tree with species tree, produced resolved alternative topologies (collapsing branches with branch supports lower than 0.95) to avoid overestimation of gene turnover and reported minimum number of gene gain and loss events.

### Tests of positive selection

To assess roles of Darwinian positive selection on the evolution of ant odorant receptor genes, we used the CodeML program from PAML package v4.8a [67] to perform positive selection tests. This program measures the nonsynonymous/synonymous rate ratio (ω = dN/dS) of the related genes. Signature of positive selection is indicated by ω > 1 [38, 68, 69]. We have two main questions in our analysis: first, did lineage-specific gene expansions evolve under positive selection, and, second, did orthologous genes that only expanded in a single or a few species evolve under positive selection compared to their orthologous genes? We used “site models” to answer the first question and “branch-site models” to answer the second question.

#### Site models

We chose lineage-specific gene clades (clades containing genes from a single species only) with bootstrap values higher than 70 % from the phylogenetic tree of hymenopteran *Or* genes (Additional file 2). We hypothesized that if these lineage-specific expansions are associated with olfactory adaptation unique to each species then positive selection should operate on these genes. The minimum number of genes per clade was set at 6 because the program cannot reliably detect selection with lower numbers of sequences, and 31 *Or* clades met these criteria.

The alignment and tree files were prepared as follows. The OR proteins from each clade were aligned using M-Coffee (http://tcoffee.crg.cat/apps/tcoffee/do:mcoffee) which finds the consensus of 4 programs (Mmafft_msa, Mmuscle_msa, Mprobcons_msa and Mt_coffee_msa). Sequences that contain large gaps (e.g. internal, N- or C-terminus missing) were removed and proteins were aligned again. All gaps in the alignment were further removed prior to the analysis using command cleandata = 1 in CodeML. These steps were done to avoid alignment error and gaps as they are known to cause false positive detection. DNA alignments were prepared using PAL2NAL and the protein alignment as a guide (http://www.bork.embl.de/pal2nal) [70]. The phylogenetic tree was made from these DNA alignments by maximum likelihood using PhyML under default parameters for a DNA tree [71].

We estimated degree of sequence divergence from mean dS values calculated under M0 (one-ratio). We limited our analysis to clades with mean dS < 1 because false positive detection could be a problem at higher sequence divergence dues to saturation of dS [36, 37]. The Log-likelihood values (*l*) were then computed under 5 different models M1a (neutral), M2a (selection), M7 (beta), M8 (beta and ω with ω ≥ 1) and M8a (beta and ω with ω = 1). Likelihood ratio tests (LRT = 2Δ*l* = 2*l*_*H1*_ − 2*l*_*H0*_) were performed to test whether the data fits the alternative model (H1) significantly better than the null model (H0) and thus indicates signature of positive selection. Pairs of alternative and null models are M8 vs. M7, M8 vs. M8a and M2a vs. M1a [68, 72, 73].

#### Branch-site models

We noticed that many orthologous genes were not in a simple 1:1 ratio. We hypothesized that genes that underwent independent lineage-specific expansion might be associated with olfactory adaptation unique to each species. Thus genes on this sub-clade (foreground branches) could have evolved under positive selection while the background branches did not. We performed this analysis on 10 *Or* clades using an updated version of the Branch-site test which allows detection of positive selection on individual amino acid residues and particular lineages [38, 69]. DNA alignment and phylogenetic trees were prepared in a similar way to the Site-test except that the foreground branches in a tree file were labeled with symbol “$1” at an ancestral node. Command settings for the alternative model was “model = 2, NSsites = 2, fix_omega = 0” and for the null model was “model = 2, NSsites = 2, fix_omega = 1, omega = 1”. To avoid convergence problems in the calculation of likelihoods, each model was run three times and the best likelihood value was kept for the LRT. LRT was then performed between the two models and compared to a Chi square distribution with 1 degree of freedom.

In cases where LRT was significant (*P* < 0.001), the Bayes Empirical Bayes (BEB) procedure was used to identify positively selected sites (PSSs) within the amino acid sequences [69]. To identify the distribution of these PSSs, they were then mapped onto the consensus receptor topology predicted by TOPCONS (http://topcons.cbr.su.se), TOPPRED (http://bioweb.pasteur.fr/seqanal/interfaces/toppred.html), and TMHMM (http://www.cbs.dtu.dk/services/TMHMM/). We used TOPO2 to generate the 2D structure diagrams of the ant odorant receptors (http://www.sacs.ucsf.edu/TOPO2).

## Additional files

Additional file 1: Dataset of candidate odorant receptor genes from the genome assemblies of Acromyrmex echinatior and Atta cephalotes identified in this study. Additional file 2: Expanded phylogenetic tree of Figure 2a. Clades used in the positive selection analyses are highlighted with color boxes (blue = Site test; yellow = Branch-site test). Additional file 3: Gene models representing each ant Or gene subfamily (Orco, A - V, and the 9-exon subfamily). Exon and intron sizes shown in the figure correspond to the actual size (average values from Or genes in the same subfamily of Atta cephalotes): black bar = Exon, line = intron, number = intron phase.

## Abbreviations

Or: odorant receptor
Orco: conserved ion co-receptor
PSSs: positively selected sites
PPs: posterior probability
