# Supplementary figures and images for: Diversification of the ant odorant receptor gene family and positive selection on candidate cuticular hydrocarbon receptors
Source: BMC Res Notes. 2015 Aug 27;8:380. doi: 10.1186/s13104-015-1371-x (PMC4549895; doi:10.1186/s13104-015-1371-x)

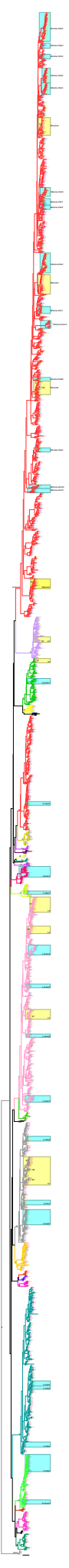

Supplement: Additional file 2: — Expanded phylogenetic tree of Figure 2a. Clades used in the positive selection analyses are highlighted with color boxes (blue = Site test; yellow = Branch-site test). [file 13104_2015_1371_MOESM2_ESM.pdf]
