# Supplementary material for: Diversification of the ant odorant receptor gene family and positive selection on candidate cuticular hydrocarbon receptors
Source: BMC Res Notes. 2015 Aug 27;8:380. doi: 10.1186/s13104-015-1371-x (PMC4549895; doi:10.1186/s13104-015-1371-x)
Supplement: Additional file 3: — Gene models representing each ant Or gene subfamily (Orco, A - V, and the 9-exon subfamily). Exon and intron sizes shown in the figure correspond to the actual size (average values from Or genes in the same subfamily of Atta cephalotes): black bar = Exon, line = intron, number = intron phase. [file 13104_2015_1371_MOESM3_ESM.pdf]

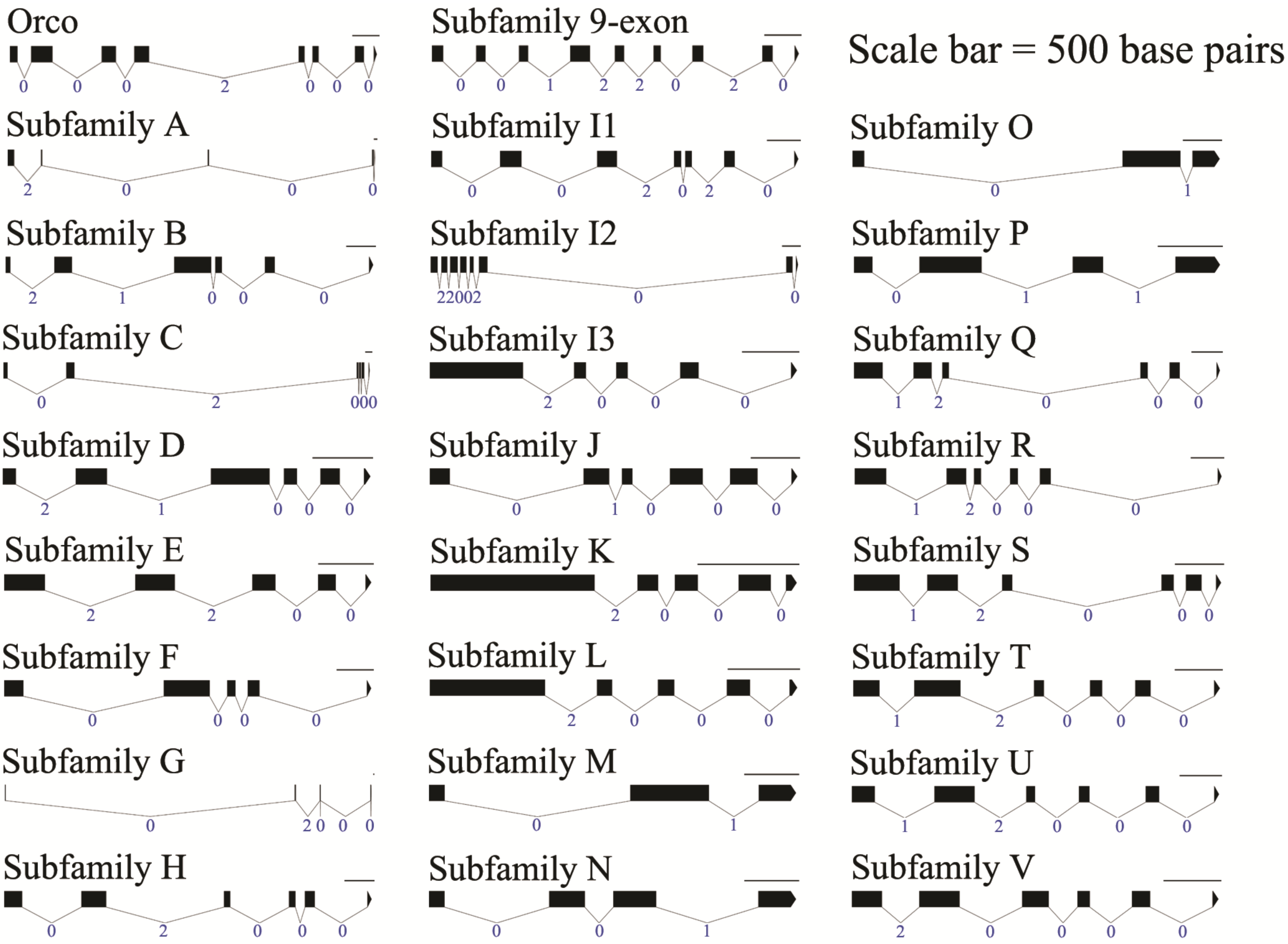

Orco
0 0 0 2 0 0 0
Subfamily A
2 0 0 0
Subfamily B
2 1 0 0 0
Subfamily C
0 2 000
Subfamily D
2 1 0 0 0
Subfamily E
2 2 0 0
Subfamily F
0 0 0 0
Subfamily G
0 2 0 0 0
Subfamily H
0 2 0 0 0
Subfamily 9-exon
0 0 1 2 2 0 2 0
Subfamily I1
0 0 2 0 2 0
Subfamily I2
22002 0 0
Subfamily I3
2 0 0 0
Subfamily J
0 1 0 0 0
Subfamily K
2 0 0 0
Subfamily L
2 0 0 0
Subfamily M
0 1
Subfamily N
0 0 1
Scale bar = 500 base pairs
Subfamily O
0 1
Subfamily P
0 1 1
Subfamily Q
1 2 0 0 0
Subfamily R
1 2 0 0 0
Subfamily S
1 2 0 0 0
Subfamily T
1 2 0 0 0
Subfamily U
1 2 0 0 0
Subfamily V
2 0 0 0 0
